# Causes of Idiocy in Massachusetts

**Published:** 1852-12

**Authors:** 


*Causes of Idiocy in Massachusetts.* — The Boston Med. and Surg. Journal, quotes from one of the Boston papers as follows: "Dr. Howe has examined carefully almost the entire number of cases of idiocy known to exist in Massachusetts, and the result is that in all but four instances, he found the parents of these idiots were intemperate, addicted to sensual vices, scrofulous, predisposed to insanity, or had intermarried with blood relations."

